# Psychometric Evaluation of the Mutuality Scale in Older Adults with Multiple Chronic Conditions and Their Caregivers Living in a Low–Middle-Income Country

**DOI:** 10.3390/nursrep16090297

**Published:** 2026-08-25

**Authors:** Dasilva Taci, Rocco Mazzotta, Manuela Saurini, Sajmira Aderaj, Alta Arapi, Alessandro Stievano, Ercole Vellone, Gennaro Rocco, Maddalena De Maria

**Affiliations:** 1 Department of Biomedicine and Prevention, University of Rome Tor Vergata, 00133 Rome, Italy; dasilva.taci@students.uniroma2.eu (D.T.); rocco.mazzotta@uniroma2.it (R.M.); ercole.vellone@uniroma2.it (E.V.); 2 Centre of Excellence for Nursing Scholarship, Order of Nurses of Rome, 00173 Rome, Italy; alessandro.stievano@unime.it (A.S.); genna.rocco@gmail.com (G.R.); 3 Faculty of Health Sciences, AAB College, 10000 Pristina, Kosovo; sajmira.aderaj@universitetiaab.com; 4 Albanian Order of Nurses, 1016 Tirana, Albania; alta_arapi@yahoo.com; 5 Department of Clinical and Experimental Medicine, University of Messina, 98100 Messina, Italy; 6 Faculty of Nursing and Midwifery, Wroclaw Medical University, 50-996 Wrocław, Poland; 7 International Center for Nursing Research Montianum, Our Lady of Good Counsel, Catholic University, 1000 Tirana, Albania; 8 Department of Life Health Sciences and Health Professions, Link Campus University, 00165 Rome, Italy; m.demaria@unilink.it

**Keywords:** older adults, mutuality scale, psychometrics, validity, confirmatory factor analysis, dyads, reliability, chronic conditions

## Abstract

**Background/Objectives**: Multiple chronic conditions (MCCs) are highly prevalent among older adults and require effective collaboration between the patient and caregiver. Mutuality, reflecting the quality of the dyadic relationship, is associated with better self-care and health outcomes. However, the Mutuality Scale (MS) has not been validated on patient–caregiver dyads managing MCCs living in a low–middle-income country (LMIC). **Aim**: This study seeks to evaluate the structural and convergent validity and reliability of the MS among patient–caregiver dyads managing MCCs living in a LMIC. **Methods**: A cross-sectional study was conducted on MCC patients and their caregiver recruited from community and outpatient settings. The MS, Self-care of Chronic Illness Inventory (SC-CII) and Caregiver Contribution to self-care Inventory (CC-SCCII) were used for measuring mutuality, patient self-care, and Caregiver Contribution (CC) to patient self-care, respectively. Confirmatory factor analysis (CFA) was performed separately for patients and caregivers to evaluate the original four-factor structure of the MS. Convergent validity was examined through correlations with self-care and CC to patient self-care. Reliability was evaluated using composite reliability and the Global Reliability Index for multidimensional scale. **Results**: A sample of 406 patient–caregiver dyads was examined. Patients had a mean age of 73.9 (±6.2) years. Caregivers had a mean age of 47.8 (±15.5) years. The four-factor structure was supported in both samples, with acceptable model fit (patients: Comparative Fit Index (CFI) = 0.953 and Root Mean Square Error of Approximation (RMSEA) = 0.078; caregiver CFI = 0.945 and RMSEA = 0.085. The second-order CFA supported a hierarchical structure. Patient and caregiver mutuality scores were strongly correlated (r = 0.778, *p* < 0.01). Higher mutuality was associated with better patient self-care (r = 0.276–0.479) and CC to self-care (r = 0.174–0.556). Reliability indices ranged from 0.70 to 0.91 for patients and 0.66 to 0.89 for caregivers. **Conclusions**: The findings support the validity and reliability of the MS for assessing mutuality in patients and their caregivers managing MCCs in an LMIC characterized by limited healthcare resources and formal support. Its use provides empirical support for assessing relationship quality and facilitating dyadic care within this vulnerable population. Future longitudinal studies should evaluate its predictive validity, responsiveness, and measurement invariance across different groups and between patient and caregiver.

## 1. Introduction

The growing number of older adults presents major challenges to health and social care systems worldwide. According to the World Health Organization (WHO) [1], by 2050, individuals aged 60 and over will comprise 22% of the global population, with nearly 80% living in low- and middle-income countries (LMICs) [1]. Older adults are increasingly affected by multiple chronic conditions (MCCs), defined as the presence of two or more chronic conditions [2]. In the United States, over 60% of adults aged 65 and older have two or more chronic conditions [1], whereas in Europe, prevalence ranges from 38% to 52%.

MCCs are also highly prevalent in LMICs [3] and are associated with poorer health outcomes compared to patients affected by a single chronic disease due to overlapping symptoms and functional limitations [3].

To address the burden of MCCs the WHO recommends involving both patients and their caregivers in the care process [4]. The Middle-Range Theory of Self-Care of Chronic Illness defines self-care as the process of maintaining health through health-promoting behaviors within the context of chronic illness management [5]. Previous studies on heart failure (HF) patients show that effective self-care is associated with better quality of life, improved survival, and lower readmission rates [6]. Importantly, self-care in MCCs is a dyadic process involving both patients and their caregivers, who contribute to care without financial compensation [7].

A central aspect of this dyadic relationship is mutuality between the patient and caregiver [8]. Numerous authors have attempted to define mutuality. Initially, Barnhill [9] introduced the concept of mutuality as “A sense of intimacy among individuals of a certain and shared identity”. Conversely, Hirschfeld [10] defined the concept of mutuality through a grounded theory as the caregiver’s ability to find gratification and meaning in the relationship with the patient. Archbold and colleagues [8] defined mutuality as “the positive quality of the relationship between caregiver and patient”. Mutuality affects the clinical outcomes of chronic patients and their relative caregivers. In patients with chronic conditions, higher mutuality is associated with a faster recovery process [11], better self-care behaviors and higher quality of life [12]. In caregivers, mutuality is associated with lower caregiver burden [13], better preparation and gratification of care [14]. In addition, previous studies highlight that when patients and caregivers perceive a high-quality relationship, they are more likely to engage effectively in the self-care process [15].

Conversely, the absence of a caregiver was found to be associated with poor patient engagement [16]. In patients, lower mutuality is associated with higher anxiety and depression [17]; in caregivers, lower mutuality is associated with higher caregiver depression, less benefit finding and more caregiving burden [18].

Mutuality is commonly assessed using the Mutuality Scale (MS), which evaluates four dimensions: love and affection, shared pleasurable activities, shared values, and reciprocity [8]. The MS has demonstrated good psychometric properties on studies conducted on patients affected by chronic conditions [19], such as HF [17], stroke [20], and coronary heart disease (CHD) [21].

The MS evaluates the quality of the patient–caregiver relationship across four theoretically grounded dimensions: love and affection, reciprocity, shared pleasurable activities, and shared values. Originally developed in English, the MS was validated on a sample of American caregivers and demonstrated high internal consistency (Cronbach’s alpha > 0.90). Specifically, Pucciarelli et al. [20] evaluated the validity of the MS on separate samples of 248 stroke survivors and 163 caregivers using confirmatory factor analysis (CFA) models, demonstrating good validity (Comparative Fit Index (CFI) = 0.94 and Root Mean Square Error of Approximation (RMSEA) = 0.06; CFI = 0.92 and RMSEA = 0.073, respectively) and high internal consistency (coefficients > 0.90 across the four dimensions for both patient and caregiver versions). Similarly, Dellafiore et al. [17] confirmed the psychometric validity of the MS on a sample of 323 HF patient–caregiver dyads, showing good validity (CFI = 0.94 and RMSEA = 0.061; CFI = 0.92 and RMSEA = 0.073, respectively) and robust internal consistency (Cronbach’s alpha = 0.94). More recently, Bassola et al. [21] tested the MS on a sample of 150 CHD patient–caregiver dyads, again confirming good factorial validity (CFI = 0.94 and RMSEA = 0.050; CFI = 0.95 and RMSEA = 0.049 on patient and caregiver samples, respectively) and adequate internal consistency (model-based internal consistency index = 0.95) [21].

Despite its importance in chronic care, the MS has not yet been validated in LMICs. Existing evidence is derived from patients with single chronic conditions living in high-income countries (HICs), where well developed healthcare systems and social support services facilitate stronger patient–caregiver relationships [22]. In contrast, LMICs frequently face limited healthcare resources, financial constraints, and restricted access to formal care, increasing caregiver burden and straining patient–caregiver relationships. These challenges may reduce mutuality, thereby compromising self-care behaviors, chronic disease management, and health outcomes [23]. Albania, a small LMIC in Southeastern Europe, has undergone substantial political and socioeconomic reforms over recent decades, accompanied by epidemiological and health system transitions that have contributed to a growing burden of chronic diseases and MCCs. Unlike HICs, where formal long-term care services are more widely available, care for people living with MCCs in Albania relies predominantly on informal family caregivers, making it a particularly relevant setting in which to examine mutuality [24]. Therefore, this study aimed to evaluate the structural validity, convergent validity and reliability of the MS among patient–caregiver dyads managing MCCs living in an LMIC.

## 2. Methods

### 2.1. Design

This is a multicenter, cross-sectional study that analyzed baseline data from an ongoing longitudinal study aimed at describing patient self-care and Caregiver Contribution (CC) to self-care in the context of MCCs [25]. The present manuscript adheres to the COnsensus-based Standards for the Selection of Health Measurement INstruments (COSMIN) guidelines [26].

### 2.2. Study Setting and Sampling

A sample of 406 patient–caregiver dyads was recruited in community and outpatient settings in northern, central and southern Albania. Patients were enrolled if they were aged 65 years or older, had a diagnosis of HF and/or diabetes mellitus (DM) and/or chronic obstructive pulmonary disease (COPD), and had at least another chronic condition. Consistent with the primary study criteria [25], patients were excluded if they presented a diagnosis of dementia and/or cancer. Caregivers were enrolled if they were aged 18 years or older and if they were identified by the patient as a primary family caregiver (unpaid family member who helps the patient in the management of the chronic conditions). Patient–caregiver dyads were excluded if either the patient or their caregiver chose not to participate. For each scale, the sample size estimation followed a commonly accepted rule of thumb, requiring a minimum of 200 individuals, which is considered sufficient for effective CFA [27].

### 2.3. Instrument

The MS [8] was used to measure mutuality from either the patient or caregiver perspective. The MS was developed in the USA and consists of 15 items grouped into four factors: (a) love and affection (items 2, 5, and 8, e.g., “How attached are you to him or her?”); (b) shared pleasurable activities (items 3, 7, 11, and 14, e.g., “How often do you like to sit and talk to him or her?”); (c) shared values (items 1 and 9, e.g., “How often do the two of you see eye to eye ?”); (d) reciprocity (items 4, 6, 10, 12, 13, and 15, e.g., “How often does he or she express feelings of appreciation for you and the things you do?”). This scale uses a five-point Likert scale ranging from 0 (“not at all”) to 4 (“a great deal”). The overall score is calculated as the average of all individual item scores, and it can range from 0 to 4, with higher scores indicating higher relationship quality. Originally developed in English, the MS was translated and culturally adapted following a standardized forward–backward translation process [28]. Two independent bilingual translators produced forward translations of the MS from English into Albanian. These versions were reconciled by consensus into a single Albanian version, which was subsequently back-translated into English by translators blind to the original instrument. The back-translated version was then compared with the original English instrument to verify conceptual, rather than literal, equivalence. Content validity and cultural relevance were assessed by three nursing experts using a Delphi survey, and the prefinal version was pilot tested with ten patient–caregiver dyads before finalization.

The Albanian version of the Self-care of Chronic Illness Inventory (SC-CII-Al) [19] was used to assess the self-care maintenance, self-care monitoring and self-care management of MCC patients. It is a theoretically grounded instrument composed of 19 items grouped in the following three scales: self-care maintenance (7 items, e.g., “Make sure to get enough sleep”), self-care monitoring (5 items, e.g., “Monitor for medication side effects”), and self-care management (7 items, e.g., “Take a medicine to make the symptom decrease or go away”). The SC-CII-Al uses a 5-point Likert scale that ranges from 1 (never) to 5 (always). Higher scores indicate better self-care, with three different 0–100 standardized scores computed for each SC-CII-Al scale. Only patients who had experienced symptoms related to their chronic conditions could complete the self-care management scale. The SC-CII-Al was validated in a previous study [19].

The Albanian version of the CC to Self-Care in Chronic Illness Inventory (CC-SC-CII-Al) [29] was used to assess the CC to patients’ self-care maintenance, self-care monitoring and self-care management. The CC-SC-CII-Al includes 19 items grouped into three distinct scales that reflect the theoretical dimensions of the CC to self-care process: CC to patients’ self-care maintenance (7 items, e.g., “Try to avoid getting sick (e.g., flu shot, wash their hands)”), CC to patients’ self-care monitoring (5 items, e.g., “Monitor the condition of the person for whom you care”), and CC to patients’ self-care management (7 items, e.g., “Think of a remedy you tried the last time the patient for whom you care had symptoms. Did the remedy make the person you care for feel better”). The CC-SC-CII-Al uses a five-point Likert scale with response options from 1 (never) to 5 (always) for CC to patients’ self-care maintenance and monitoring and from 1 (not likely) to 5 (very likely) for CC to patients’ self-care management. Each scale generates a standardized score from 0 to 100, where higher scores reflect better CC to self-care.

The sociodemographic characteristics (i.e., age, gender, marital status, family income, level of education, and relationship between patient and caregiver) of patient–caregiver dyads were collected using an *ad hoc* instrument developed for the study. The clinical documentation was also consulted for specific patient clinical information (e.g., number and type of chronic conditions).

### 2.4. Data Collection

Trained nurse researchers collected data from September 2020 until August 2024 through face-to-face interviews. Patients identified as eligible were invited to participate after the study objectives and data collection procedures were explained to them.

### 2.5. Statistical Analysis

The analysis of descriptive statistics was conducted using IBM SPSS Statistics (Version 26; IBM Corp., Armonk, NY, USA), while factor analyses were conducted in Mplus (Version 8.4).

Sociodemographic characteristics and responses to the Mutuality Scale items for both patients and their caregivers were described using descriptive statistics, including measures of central tendency (mean and median), dispersion (standard deviation), and frequency distributions. To evaluate the normality of the MS items, skewness and kurtosis univariate indices were considered.

Consistent with the psychometric literature, the analysis proceeded with an examination of the MS factorial structure on patients and on caregivers, followed by an evaluation of its reliability [30]. In line with the original development study by Archbold et al. [8], a CFA was conducted to examine the factorial structure (dimensionality) of the MS. According to previous studies [17,20,21], separate CFAs were performed for the patient and caregiver samples, referred to as Mutuality Patients and Mutuality Caregivers, respectively. In both samples, a four-factor model was tested, comprising: love and affection (items #2, #5, and #8), shared pleasurable activities (items #3, #7, #11, and #14), shared values (items #1 and #9), and reciprocity (items #4, #6, #10, #12, #13, and #15). To account for the correlations among the four latent factors, a second-order factor model was also tested. The non-normal distribution of the MS item scores is recommended for handling continuous or approximately continuous indicators with non-normal distributions by providing robust standard errors and a scaled chi-square statistic [31,32]. This approach is consistent with Van der Linden’s recommendations and with previous psychometric validation studies of the MS [20,21,33]. Model adequacy was assessed through a multifaceted evaluation strategy incorporating several goodness-of-fit indices following established recommendations: the CFI, the Tucker–Lewis Index (TLI), the RMSEA, and the Standardized Root Mean Square Residual (SRMR) [34]. Fit criteria were defined as follows: CFI and TLI values equal to or above 0.95 indicated excellent model fit, while values between 0.90 and 0.95 were deemed acceptable [35]. For the RMSEA, values ≤ 0.05 suggested a close fit [36], those between 0.05 and 0.08 reflected reasonable approximation [37], and values ≥ 0.10 indicated inadequate fit [38]. RMSEA confidence intervals (90% CI between ≤0.05 and ≤0.08), together with *p*-values > 0.05, were also considered indicative of acceptable fit [39]. Similarly, SRMR values ≤ 0.08 were interpreted as evidence of satisfactory model fit [40]. The chi-square statistics (χ^2^) were also computed and interpreted together with the above indices. To assess the stability of the factor models, a non-parametric bootstrap procedure (5000 resamples) was performed on the final models, and 95% confidence intervals were examined for the model parameter estimates.

Reliability, in terms of internal consistency, was examined using multiple indices tailored to the factorial structure of each scale. For the patient and caregiver Mutuality Scales, reliability estimates were derived from model parameters and quantified using the composite reliability coefficient, in line with Fornell and Larcker’s [41] approach. Furthermore, to account for scale multidimensionality, the Global Reliability Index for multidimensional scale was tested [42]. A threshold of 0.70 or higher was adopted as the criterion for acceptable internal consistency [43]. In addition, we computed the item-total corrected correlations, which should have a value of 0.30 or greater [44].

The responsiveness to change, as an expression of the measurement precision of the Mutuality Patients and Mutuality Caregivers samples, was assessed through the estimation of the standard error of measurement (SEM) and the smallest detectable change (SDC). In light of the instrument’s four-dimensional structure, SEM was calculated using model-based reliability coefficients, applying the formula SD × √(1 − reliability coefficient), as outlined by Brown [45]. Lower values of the SEM and SDC were interpreted as indicative of greater precision in detecting change.

The hypotheses for testing the construct validity were theoretically grounded in Interdependence Theory [46] and the Dyadic Illness Management model [47], which conceptualize chronic illness management as a shared, relational process within the patient–caregiver dyad using Pearson’s correlation coefficient. We used Cohen’s recommendations to judge the effect size of the correlations [48]. Briefly, values ranging from 0.10 to 0.29 indicated weak correlations, from 0.30 to 0.50 indicated moderate correlations, and values greater than 0.50 indicated strong correlations. Three hypotheses were formulated: (1) that patients’ and caregivers’ scores on the MS would be positively and significantly correlated, reflecting interdependence in their perceptions of mutuality within the caregiving relationship [47]; (2) that patients’ MS scores would be positively and significantly associated with self-care behaviors, indicating that higher perceived mutuality is related to greater engagement in self-care [12,13,17]; and (3) that caregivers’ MS scores would be positively and significantly associated with the CC to patient self-care maintenance, monitoring, and management scores [49].

The floor/ceiling effect for the MS was analyzed to assess the interpretability, which was calculated as the percentage of participants scoring at the bottom and top of the scale. As suggested by COSMIN consensus [50], fewer than 15% of responses with either the lowest (score = 0) or the highest score (score = 4) were deemed acceptable, indicating negligible floor and ceiling effects.

### 2.6. Ethical Consideration

Ethical approval for the study was obtained from the Catholic University of Our Lady of Good Counsel with protocol number 237/2020. The study protocol adheres to the principles outlined in the Helsinki Declaration and was crafted with utmost consideration for the rights of the participants. Data were treated with confidentiality, and participants provided written informed consent. Those who declined to provide informed consent were excluded from the study. To ensure participant anonymity, identification codes were assigned upon enrollment.

## 3. Results

### 3.1. Characteristics of the Sample

The sociodemographic characteristics of patients with chronic conditions and their caregivers are presented in Table 1. A sample of 406 MCC patient–caregiver dyads was enrolled. Patients had a mean age of 73.9 (SD = 6.2) years: 53.7% were female, and 64.0% had a low educational level (0–8 years). Most (68.7%) patients were married and perceived their income as sufficient for living (75.6%). The mean number of chronic conditions was (2.5, SD = 0.7), with hypertension (86.9%) and DM (74.6%) being the most prevalent.

The caregivers had a mean age of 47.8 years (SD = 15.5) and were predominantly female (67.5%). More than one-third (37.6%) of caregivers were the patients’ children. Approximately 81% of caregivers reported being employed, and over half (59.1%) lived with patients. Caregivers provided an average of 21.8 h of assistance per week and had been involved in caregiving for an average duration of 6.4 years.

### 3.2. Item Descriptions Mutuality Scale

Item descriptions for the patient and caregiver versions of the MS, including means, standard deviations, skewness, kurtosis, and corrected item-total correlation, are presented in Table 2. For patients, the MS items with the highest scores were reported for item #8, “How much love do you feel for him or her,” and item #12, “How often do you confide in him or her.” The items with the lowest scores were observed for item #11, “How often do the two of you laugh together,” and item #15, “How often does he or she express feelings of warmth toward you.”

Regarding caregivers, the highest scores were reported for item #5, “How attached are you to him or her,” and item #8, “How much love do you feel for him or her”, while the lowest scores were observed for items #6, “How often does he or she help you,” and #11, “How often do the two of you laugh together.” Most items were normally distributed, showing no excessive skewness or kurtosis.

### 3.3. Factorial Structure of the Mutuality Scale

#### 3.3.1. Dimensionality of the Patient Version of the Mutuality Scale

The four-factor model, comprising the dimensions of love and affection, shared pleasurable activities, shared values, and reciprocity, was tested. This model had a partially adequate fit: χ^2^(84, N = 406) = 325.239, *p* < 0.001; CFI = 0.944; TLI = 0.930; RMSEA = 0.084 (90% CI = 0.075–0.094, *p* < 0.001); and SRMR = 0.037. However, excessive covariance was observed between items #2 (“How often do you feel physically close to him or her?”) and #3 (How often do you enjoy sharing past experiences with him or her?”) as well as between items #2 and #4 (“How often does he or she express feelings of appreciation for you and the things you do?”). This issue may be explained by the proximity of the items within the scale, which could have inflated the shared covariance, a phenomenon known as the “proximity effect” [51]. Methodologically, when items are physically adjacent, participants often exhibit a response consistency that inflates error covariances beyond the latent construct, necessitating these model adjustments to ensure a more accurate representation of the factor structure [35,42]. Another explanation could be that although all three items reflect the broader construct of mutuality, item #2 specifically captures the experience of emotional and physical closeness within the relationship. Such closeness may naturally facilitate the sharing of meaningful life experiences and the expression of appreciation, resulting in shared variance beyond that explained by the latent factor alone. In addition, the observed residual covariance may also be attributable to similarities in item wording and overlapping conceptual content, which can generate local item dependence without necessarily indicating misspecification of the latent construct.

Consistent with the recommendations provided by Bagozzi et al. [43] and Fornell et al. [41], these covariances were explicitly modeled, leading to an improvement in fit: χ^2^(82, N = 406) = 285.411, *p* < 0.001; CFI = 0.953; TLI = 0.940; RMSEA = 0.078 (90% CI = 0.068–0.088, *p* < 0.001); and SRMR = 0.036. All factor loadings were statistically significant and greater than 0.677.

Given the high correlations among the four latent factors (ranging from 0.675 to 0.915), a second-order factor model was subsequently tested. This model also demonstrated acceptable fit indices: χ^2^(84, N = 406) = 292.986, *p* < 0.001; CFI = 0.952; TLI = 0.940; RMSEA = 0.078 (90% CI = 0.069–0.088, *p* < 0.001); and SRMR = 0.037.

**Figure 1:**
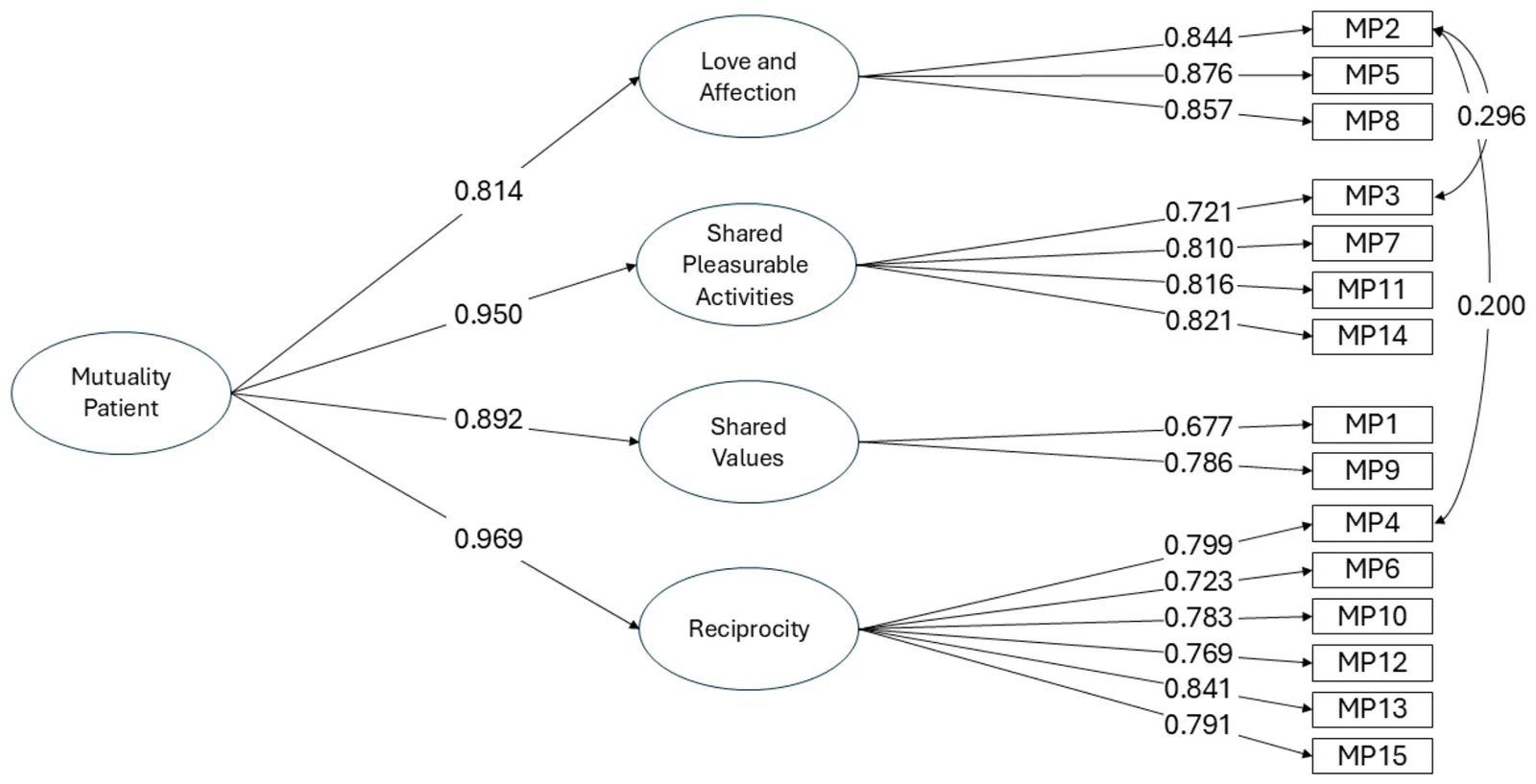
Confirmatory factor analysis of the Mutuality Scale of the patient version (N = 406 patients). Note. The results are derived from Mplus fully standardized solutions, with all coefficients reaching statistical significance (*p* < 0.05). The values shown next to the single-headed arrows represent factor loadings, while the values next to the double-headed arrows indicate correlation coefficients.

Overall, these results support a four-factor structure at the first-order level and indicate the presence of a hierarchical unidimensional construct, as illustrated in Figure 1. Finally, bootstrap resampling confirmed the stability of the model parameters, including the factor loadings and the residual covariances specified through modification indices (Table S1).

#### 3.3.2. Dimensionality of the Mutuality Scale of the Caregiver Version

The four-factor model originally hypothesized for the patient sample was also tested in the caregiver sample and yielded inadequate goodness-of-fit indices: χ^2^(84, N = 406) = 406.815, *p* < 0.001; CFI = 0.922; TLI = 0.903; RMSEA = 0.097 (90% CI = 0.088–0.094, *p* < 0.107); and SRMR = 0.045.

Inspection of the parameter estimates revealed that one of the residual covariances found in Dellafiore et al. [17] resulted in a significant relationship, in particular, items #4 (“How often does he or she express feelings of appreciation for you and the things you do?”) and #15 (“How often does he or she express feelings of warmth toward you?”).

However, several item pairs exhibited excessive covariance, specifically between: items #2 (“How often do you feel physically close to him or her?”) and #3 (“How often do you enjoy sharing past experiences with him or her?”); items #3 and #5 (“How attached are you to him or her?”); items #1 (“How often do the two of you see eye to eye?”) and #4; and items #15 and #11 (“How often do the two of you laugh together?”). As observed in the patient sample, the covariances detected in the caregiver data may be attributed to the close proximity of specific items within the scale [51], as well as by similarities in item wording and overlapping conceptual content, which may have generated shared residual variance beyond that accounted for by the latent factor.

The identified covariances were specified in the model, resulting in a notable improvement in fit indices: χ^2^(79, N = 406) = 309.070, *p* < 0.001; CFI = 0.945; TLI = 0.926; RMSEA = 0.085 (90% CI = 0.075–0.095, *p* < 0.001); and SRMR = 0.042. All factor loadings were statistically significant, with values exceeding 0.649. Given the strong correlations among the four factors (ranging from 0.701 to 0.931), a second-order factor model was subsequently evaluated. This model also demonstrated acceptable fit: χ^2^(81, N = 406) = 309.745, *p* < 0.001; CFI = 0.945; TLI = 0.929; RMSEA = 0.083 (90% CI = 0.074–0.093, *p* < 0.001); and SRMR = 0.042.

**Figure 2:**
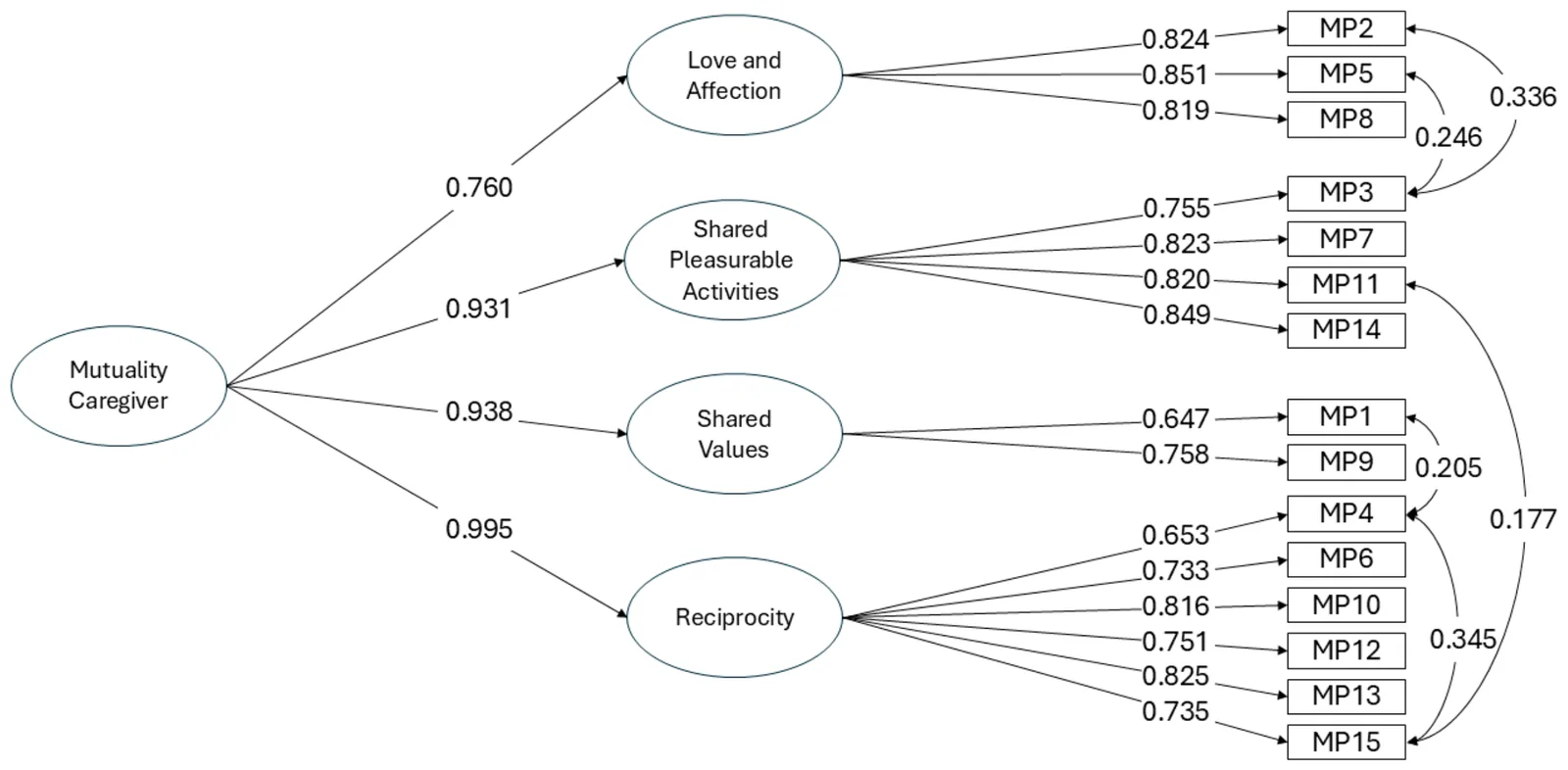
Confirmatory factor analysis of the Mutuality Scale (N = 406 caregivers). Note. The results are derived from Mplus fully standardized solutions, with all coefficients reaching statistical significance (*p* < 0.05). The values shown next to the single-headed arrows represent factor loadings, while the values next to the double-headed arrows indicate correlation coefficients.

These findings provide empirical support for the factorial validity of the MS of the caregiver version, confirming a four-factor structure at the first-order level and the presence of a hierarchical second-order model (Figure 2). Finally, bootstrap resampling confirmed the stability of the model parameters, including the factor loadings and all five residual covariances specified through modification indices (Table S1).

### 3.4. Reliability and Measurement Errors of the Mutuality Scale

Composite reliability coefficients supported the internal consistency of the MS in both patients and caregivers. In the patient version, coefficients ranged from 0.70 to 0.91 across the first-order factors. The Global Reliability Index for the second-order factor was also high (0.91), indicating strong model-based internal consistency. Measurement error was acceptable, with SEM and SDC values supporting good precision of the Mutuality Scale.

Similarly, for the caregiver version, composite reliability coefficients ranged from 0.66 to 0.89. Notably, the ‘shared values’ factor did not meet the conventional threshold for internal consistency, showing a coefficient of 0.66. However, the second-order factor demonstrated excellent internal consistency, with a Global Reliability Index equal to 0.90. As with the patient version, SEM and SDC values confirmed adequate measurement precision for the mutuality construct in caregivers. Table 3 presents the reliability indices for first- and second-order factors, as well as SEM and SDC values for both versions of the MS. Finally, all items had adequate discrimination, with a corrected item-total correlation greater than 0.30 in both the patient and caregiver versions (Table 2).

### 3.5. Convergent Validity

We tested the hypothesis that the scores of the MS, including each of its factors, would be positively and significantly correlated across the patient and caregiver. The results supported this hypothesis, showing moderate to very strong positive correlations between patient and caregiver MS scores (Pearson’s r ranged from 0.530 to 0.950 and from 0.538 to 0.954, *p* < 0.01, in patients and caregivers, respectively). Furthermore, a strong correlation was observed between the total MS scores reported by patients and caregivers (r = 0.778, *p* < 0.01).

It was also hypothesized that patient MS scores would be positively and significantly associated with the three dimensions of self-care (self-care maintenance, self-care monitoring and self-care management). The results confirmed this hypothesis, revealing small-to-moderate positive correlations between MS scores and all self-care dimensions (Pearson’s r ranged from 0.276 to 0.479, *p* < 0.01). Although these associations were statistically significant, their magnitude was low to moderate. This indicates that while mutuality is a relevant correlate of self-care, it represents only one of the complex factors influencing illness management in this population, maintaining the scientific objectivity of the findings.

Finally, we hypothesized that the caregiver MS scores would be positively and significantly associated with the CC to patient self-care. This hypothesis was also confirmed, showing small to moderate positive correlations (Pearson’s r ranged from 0.174 to 0.556, *p* < 0.01).

Table 4 presents the correlation coefficients between MS scores (total, patient and caregiver) and the three dimensions of self-care and of CC to patient self-care.

### 3.6. Floor/Ceiling Effect

No floor effects were observed for either version of the Mutuality Scale, as no participant achieved the minimum possible total score. Ceiling effects were 12.1% for the patient version and 9.9% for the caregiver version.

## 4. Discussion

The aim of this study was to test the measurement properties of the MS in patients with MCCs and their caregivers. Specifically, the study provided evidence of structural and convergent validity and reliability of the MS among patient–caregiver dyads managing MCCs living in an LMIC. To our knowledge, this is the first study that tested the measurement properties of the MS in patients with MCCs and their caregivers living in settings with limited healthcare resources and formal support. Previous validation studies were conducted exclusively in HICs and among patients with a single chronic condition. The factor structure identified in our sample replicated the structure previously identified in patients and caregivers in contexts of HF [17], stroke [20] and CHD [21]. Consistent with the theoretical framework proposed by Archbold et al. [8], CFA confirmed the original four-factor structure, indicating that the components of mutuality (love, shared pleasurable activities, shared values, and reciprocity) are similarly perceived by patient–caregiver dyads in this LMIC context. Our findings support the use of the MS as a valid and reliable instrument for assessing mutuality and advancing research on its determinants and outcomes across the trajectory of chronic disease in LMICs.

Regarding factorial validity, two residual covariances were specified for the patient version of the MS (items #2–#3 and #2–#4), whereas five were required for the caregiver version (items #2–#3, #3–#5, #11–#15, #1–#4, and #4–#15). These correlated residuals involved conceptually related items reflecting emotional closeness, appreciation, warmth, and shared experiences, suggesting the presence of relational processes not fully explained by the latent mutuality construct [52]. Such associations are likely attributable to local item dependence arising from overlapping interpersonal aspects of mutuality rather than misspecification of the underlying factorial structure. However, because these residual covariances were specified based on modification indices, they may partially reflect sample-specific characteristics. Consequently, although theoretically justified, their inclusion may reduce the generalizability of the model to other populations and should therefore be interpreted cautiously. Future studies should examine whether these correlated residuals are replicated in independent samples and across different cultural contexts. In the caregiver sample, although the RMSEA value (0.085; 90% CI = 0.075–0.095, *p* < 0.001) slightly exceeded the conventional threshold for close model fit, the overall fit of the model remained acceptable when considered alongside the other fit indices.

The results of hypothesis testing supported the convergent validity of the MS. Consistent with findings reported in HICs [17], mutuality scores in our sample were significantly associated with self-care behaviors and CC to patient self-care in all three theoretical dimensions. The modest-to-moderate correlations observed between mutuality and self-care dimensions, while supporting convergent validity, also suggest that mutuality alone does not fully account for self-care behaviors. This is consistent with multidimensional models of self-care, suggesting that mutuality represents an important relational resource supporting self-care, alongside other individual, interpersonal, and contextual determinants. From a clinical perspective, these findings suggest that assessing mutuality may help clinicians identify patient–caregiver dyads who may experience difficulties in collaborative self-care. Recognizing lower levels of mutuality could inform targeted dyadic interventions aimed at strengthening the patient–caregiver relationship and improving chronic disease management.

To assess the reliability of the MS, each dimension was evaluated using composite reliability coefficients, which provide a more robust estimation of internal consistency in latent variable models. All subscales demonstrated acceptable internal consistency, with composite reliability coefficients exceeding the recommended threshold of 0.70 except for the shared values factor. The slightly lower reliability observed for this subscale among caregivers (ω = 0.66) should be interpreted in the context of its brevity, as it comprises only two items. Reliability coefficients are inherently influenced by the number of items in a scale, and short subscales commonly yield lower estimates despite adequate conceptual coherence and content validity. Nevertheless, if subscale scores are to be used independently, particular attention should be paid to the psychometric properties of the shared values factor. Variability in the perception and expression of cultural differences within patient–caregiver relationships may partly explain the lower reliability observed in this population. Although the MS is most commonly applied using the total score, future research should explore the distinct contribution of each factor in relation to relevant predictors and outcomes. Such an approach would enhance understanding of the scale’s construct validity and its application across different populations, particularly of LMICs. The model-based reliability index for the overall scale further supported the adequacy of internal consistency across both patient and caregiver versions.

The low values of the SEM and SDC provide evidence of the scale’s measurement precision and support its clinical applicability. The absence of floor effects and the negligible ceiling effects observed in both patients and caregivers further support the interpretability of the MS, indicating adequate discriminative capacity across the range of scores without excessive clustering at the extremes. These findings indicate that the MS is capable of detecting meaningful changes in mutuality over time that are unlikely to be attributable to measurement error, making it a valuable instrument for monitoring patient–caregiver relationships and evaluating the effectiveness of interventions aimed at improving dyadic functioning. Regarding score interpretation, higher MS scores reflect higher perceived mutuality within the patient–caregiver dyad, facilitating the assessment of this important relational construct. Although clinically validated cutoff values are not currently available, continuous scores may provide useful insights into the quality of the dyadic relationship. Specifically, higher scores may reflect stronger relational support and dyadic coping, whereas lower scores may indicate potentially vulnerable dyads experiencing difficulties in collaborative self-care or greater caregiver strain. Therefore, MS scores should be evaluated along a continuum of relationship quality to guide targeted dyadic interventions rather than using categorical clinical thresholds.

This study has some limitations. First, although the study was multicentric, recruitment was conducted in one LMIC and relied on convenience sampling. These factors may limit the external validity and generalizability of our findings to LMICs, cultures, settings, individuals with other combinations of chronic conditions and other socioeconomic contexts. To mitigate this limitation, we recruited participants from all Albania regions, thereby enhancing the geographic and sociodemographic diversity of the sample. Nevertheless, future studies employing probability-based sampling and more representative populations are needed to confirm these findings and strengthen their generalizability. Second, the cross-sectional design represents a major methodological limitation, as it precludes the assessment of the predictive validity and longitudinal stability of the MS over time. Consequently, the ability of the instrument to predict clinically relevant outcomes and to detect changes in mutuality across the caregiving trajectory has not been examined. Future longitudinal studies should investigate the test–retest stability, predictive validity (assessing caregiver burden, hospitalization, long-term self-care outcomes, etc.), and longitudinal responsiveness of the MS to determine its suitability for monitoring changes in mutuality over time and for predicting relevant psychosocial and clinical outcomes. Third, patients with dementia and cancer were excluded from the study; therefore, the findings cannot be generalized to these populations. Fourth, our sample exhibited an uneven sex distribution for both patients and caregivers (mostly female). It is possible that the results might be different in a sample with different sex distributions. However, this demographic distribution reflects the population of patients in LMICs with MCCs and their caregivers. Future studies should examine measurement equivalence across patient and caregiver, cross-cultural and gender groups to determine whether the MS operates equivalently across informants, cultural contexts and gender. Fifth, discriminant validity and test–retest reliability were not assessed. Future research should evaluate whether the MS can be empirically distinguished from related constructs and the temporal stability of the MS. Although data were collected through face-to-face interviews rather than self-administered questionnaires, reducing some sources of common method bias typical of self-report surveys, the use of a single assessment session and a single interviewer per participant does not fully rule out shared-method influences (e.g., social desirability and interviewer effects). Future studies could benefit from integrating interview-based data with independently collected or objective measures.

In addition, further research should evaluate the measurement invariance between patients and caregivers, which are essential for meaningful comparisons of their latent mutuality scores, and across relevant demographic and clinical subgroups, using a dyadic approach.

A key strength of the study lies in the large sample of patient–caregiver dyads with a very low rate of missing data. This was likely facilitated by face-to-face data collection, during which trained nurse researchers guided participants through the assessment and provided clarification when needed, thereby reducing the likelihood of incomplete responses [53,54].

The MS provides nurses with a reliable instrument to support the transition from an individual- to a dyadic-centered model of care in LMICs. By integrating this MS into routine clinical practice, nurses can proactively identify patient–caregiver dyads with low levels of mutuality, which often can compromise effective chronic disease management and caregiver well-being. Early identification of vulnerable patient–caregiver dyads can facilitate the implementation of targeted interventions (as communication coaching, relationship-focused education, shared problem-solving, caregiver support strategies, etc.) with the aim of strengthening patient–caregiver dyad functioning and promoting collaborative chronic disease management.

Furthermore, these findings provide the evidence of a valid and reliable instrument for assessing mutuality among patient–caregiver dyads in resource-constrained LMICs, where optimizing limited healthcare resources and strengthening informal caregiving are essential for effective chronic disease management. Future studies should use the MS to investigate the trajectory of mutuality over time, examine its determinants, and evaluate its associations with clinically relevant outcomes.

## 5. Conclusions

Our study provides empirical support for the use of the MS in patient–caregiver dyads managing MCCs in LMICs, supporting its applicability in assessing relationship quality within this vulnerable population. These findings contribute to the limited evidence on mutuality in this context and offer clinicians a validated tool to identify dyads at risk of poor relationship quality. Future research should further explore how mutuality interacts with other psychosocial factors across diverse LMIC settings, examine its longitudinal relationship with patient and caregiver health outcomes and test cross-informant measurement invariance between patient and caregiver versions of the MS.

## Figures and Tables

**Table 1:** Sociodemographic and clinical characteristics of MCCs patients (*n* = 406) and their caregivers (*n* = 406).

Variable	Patient	Caregiver
	Mean (SD)	Mean (SD)
Age (years)	73.9 (6.2)	47.8 (15.5)
	N (%)	N (%)
Gender		
Male	188 (46.3)	132 (32.5)
Female	218 (53.7)	274 (67.5)
Marital status		
Married/partnered	279 (68.7)	320 (78.8)
Single		73 (18.0)
Widow/divorced	123 (30.3)	12 (2.9)
Education		
0–8 years	260 (64.0)	122 (30.0)
≥9 years	146 (35.9)	284 (69.9)
Relationship to patient		
Son/daughter		153 (37.6)
Husband/wife		101 (24.8)
Other		150 (36.9)
Employment status		
Employed	11 (2.7)	325 (80.6)
Unemployed/retired	395 (97.3)	78 (19.2)
Income		
More than needed	20 (4.9)	44 (10.8)
Enough for living	307 (75.6)	309 (76.1)
Less than needed	79 (19.5)	49 (12.1)
Chronic conditions		
Hypertension	353 (86.9)	
Diabetes	303 (74.6)	
Heart failure	97 (23.9)	
COPD	54 (13.3)	
Osteoporosis	47 (11.5)	
Arthritis	46 (11.3)	
Other	62 (15.2)	
	Mean, (SD), Median (Q1–Q3)	N (%)
Caregiver living with patient		240 (59.1)
Number of chronic conditions	2.5 (0.7), 2.0 (2.0–3.0)	
Hours of caregiving	21.8 (9.9), 20.0 (14.0–28.0)	
Years of caregiving	6.4 (3.9), 5.0 (3.0–8.0)	

Legend. N, number of participants; %, percentage; SD, standard deviation; COPD, chronic obstructive pulmonary disease; Q1, first quartile; Q3, third quartile.

**Table 2:** Mutuality Scale item descriptive analyses.

	Patients			Caregivers		
Item	Mean (SD)	Skewness (Kurtosis)	Corrected Item-Total Correlation	Mean (SD)	Skewness (Kurtosis)	Corrected Item-Total Correlation
1. How often do the two of you see eye to eye?	2.99 (0.81)	−0.43 (−0.35)	0.590	2.91 (0.87)	−0.46 (−0.24)	0.604
2. How often do you feel physically close to him or her?	3.40 (0.69)	−0.91 (−0.04)	0.708	3.40 (0.73)	−1.01 (0.66)	0.661
3. How often do you enjoy sharing past experiences with him or her?	3.07 (0.80)	−0.50 (−0.33)	0.667	2.95 (0.85)	−0.38 (−0.49)	0.722
4. How often does he or she express feelings of appreciation for you and the things you do?	3.17 (0.80)	−0.69 (0.21)	0.776	3.21 (0.86)	−0.96 (0.60)	0.673
5. How attached are you to him or her?	3.43 (0.74)	−1.09 (0.69)	0.727	3.42 (0.74)	−1.14 (1.00)	0.671
6. How often does he or she helps you?	3.35 (0.74)	−1.10 (1.09)	0.679	2.69 (1.00)	−0.55 (0.04)	0.675
7. How often do you like to sit and talk to him or her?	3.17 (0. 73)	−0.43 (−0.52)	0.758	3.00 (0.79)	−0.31 (−0.61)	0.76
8. How much love do you feel for him or her?	3.52 (0.67)	−1.18 (0.53)	0.717	3.51 (0.71)	−1.27 (0.78)	0.655
9. To what extent do the two of you share the same values?	3.01 (0.81)	−0.41 (−0.49)	0.678	3.02 (0.85)	−0.43 (−0.68)	0.676
10. When you really need it, how much does he or she comfort you?	3.29 (0.75)	−0.73 (0.04)	0.733	3.04 (0.85)	−0.72 (0.28)	0.769
11. How often do the two of you laugh together?	2.86 (0.81)	−0.16 (−0.52)	0.759	2.88 (0.79)	−0.05 (−0.85)	0.769
12. How often do you confide in him or her?	3.53 (0.63)	−1.15 (0.74)	0.739	3.40 (0.68)	−0.80 (−0.15)	0.727
13. How much emotional support does he or she give to you?	3.26 (0.71)	−0.68 (−0.38)	0.778	3.05 (0.86)	−0.66 (0.18)	0.772
14. To what extent do you enjoy the time the two of you spend together?	3.26 (0.69)	−0.47 (−0.46)	0.774	3.11 (0.73)	−0.29 (−0.70)	0.768
15. How often does he or she express feelings of warmth toward you?	2.94 (0.95)	−0.42 (−0.73)	0.756	3.03 (0.96)	−0.68 (−0.28)	0.727

Legend. SD, standard deviation.

**Table 3:** Reliability coefficients and measurement errors of the Mutuality Scale of patients (*n* = 406) and their caregivers (*n* = 406).

	Patient	Caregiver
Composite reliability coefficients		
Love	0.89	0.87
Shared pleasurable activities	0.87	0.89
Shared values	0.70	0.66
Reciprocity	0.91	0.89
Global reliability index		
Total score	0.91	0.90
Measurement Errors	SEM (SDC)	SEM (SDC)
Total score	0.17 (0.48)	0.19 (0.53)

Legend. SEM, standard error of measurement; SDC, smallest detectable change.

**Table 4:** Bivariate correlation of construct validity of MS.

Variable	Love and Affection	Shared Pleasurable Activities	Shared Values	Reciprocity	Self-Care Maintenance	Self-Care Monitoring	Self-Care Management
MS patient version							
Love and affection	-				0.301	0.306	0.276
Shared pleasurable activities	0.695				0.469	0.456	0.388
Shared values	0.530	0.683			0.450	0.453	0.350
Reciprocity	0.723	0.804	0.691		0.454	0.397	0.331
Total score	0.831	0.915	0.786	0.950	0.479	0.451	0.382
					CC maintenance	CC monitoring	CC management
MS caregiver version							
Love and affection	-				0.325	0.364	0.174
Shared pleasurable activities	0.647				0.447	0.507	0.353
Shared values	0.538	0.691			0.465	0.527	0.403
Reciprocity	0.669	0.827	0.714		0.528	0.538	0.308
Total score	0.791	0.918	0.803	0.954	0.515	0.556	0.349
The correlation between the scores of MS patient and MS caregiver is equal to 0.778 (*p* < 0.01)							

Legend. MS, Mutuality Scale; CC, Caregiver Contribution. Note. Self-care behaviors were measured by Self-care of Chronic Illness Inventory; CC to patient self-care behaviors were measured by CC to Self-Care of Chronic Illness Inventory. All correlations were significant at the 0.01 level (two-tailed).

## Data Availability

The dataset is available upon request from the authors.

## Supplementary Materials

The following supporting information can be downloaded at https://www.mdpi.com/article/10.3390/nursrep16090297/s1, Table S1: Bootstrap confidence intervals (5000 resamples) for the standardized factor loadings and residual covariances of the final Mutuality Scale models (patients and caregivers).

## Public Involvement Statement

No public involvement in any aspect of this research is reported.

## Guidelines and Standards Statement

This manuscript was drafted against the STROBE Statement for cross-sectional studies.

## References

[1] World Health Organization (2025). Ageing and Health.

[2] Dattalo M., DuGoff E., Ronk K., Kennelty K., Gilmore-Bykovskyi A., Kind A.J. (2017). Apples and Oranges: Four Definitions of Multiple Chronic Conditions and their Relationship to 30-Day Hospital Readmission. J. Am. Geriatr. Soc..

[3] Kaluvu L., Asogwa O.A., Marza-Florensa A., Kyobutungi C., Levitt N.S., Boateng D., Klipstein-Grobusch K. (2022). Multimorbidity of communicable and non-communicable diseases in low- and middle-income countries: A systematic review. J. Multimorb. Comorbidity.

[4] World Health Organization (2022). WHO Guideline on Self-Care Interventions for Health and Well-Being, 2022 Revision.

[5] Riegel B., Jaarsma T., Strömberg A. (2012). A middle-range theory of self-care of chronic illness. Adv. Nurs. Sci..

[6] Jaarsma T., Hill L., Bayes-Genis A., La Rocca H.B., Castiello T., Celutkiene J., Marques-Sule E., Plymen C.M., Piper S.E., Riegel B. (2021). Self-care of heart failure patients: Practical management recommendations from the Heart Failure Association of the European Society of Cardiology. Eur. J. Heart Fail..

[7] De Maria M., Ausili D., Lorini S., Vellone E., Riegel B., Matarese M. (2022). Patient Self-Care and Caregiver Contribution to Patient Self-Care of Chronic Conditions: What Is Dyadic and What It Is Not. Value Health.

[8] Archbold P.G., Stewart B.J., Greenlick M.R., Harvath T. (1990). Mutuality and preparedness as predictors of caregiver role strain. Res. Nurs. Health.

[9] Barnhill L.R. (1979). Healthy family systems. Fam. Coord..

[10] Hirschfeld M. (1983). Homecare versus institutionalization: Family caregiving and senile brain disease. Int. J. Nurs. Stud..

[11] Park E.O., Schumacher K.L. (2014). The state of the science of family caregiver-care receiver mutuality: A systematic review. Nurs. Inq..

[12] Vellone E., Dellafiore F., Chung M.L., Alvaro R. (2018). Mutuality and self-care in heart failure patient and caregiver dyads. Eur. J. Cardiovasc. Nurs..

[13] Hooker S.A., Schmiege S.J., Trivedi R.B., Amoyal N.R., Bekelman D.B. (2018). Mutuality and heart failure self-care in patients and their informal caregivers. Eur. J. Cardiovasc. Nurs..

[14] Simeone S., Savini S., Torino F., Vellone E., Alvaro R. (2014). Mutuality in caregiving: A literature review. Prof. Inferm..

[15] Riegel B., Dunbar S.B., Fitzsimons D., Freedland K.E., Lee C.S., Middleton S., Stromberg A., Vellone E., Webber D.E., Jaarsma T. (2021). Self-care research: Where are we now? Where are we going?. Int. J. Nurs. Stud..

[16] Salari A., Rouhi Balasi L., Ashouri A., Moaddab F., Zaersabet F., Nourisaeed A. (2018). Medication Adherence and its Related Factors in Patients Undergoing Coronary Artery Angioplasty. J. Caring Sci..

[17] Dellafiore F., Buck H.G., Pucciarelli G., Barbaranelli C., Paturzo M., Alvaro R., Vellone E. (2018). Psychometric characteristics of the mutuality scale in heart failure patients and caregivers. Heart Lung.

[18] Glickman A., Mikulich-Gilbertson S., Abshire Saylor M., DeGroot L., Bekelman D.B. (2025). Relationship Status and Quality Are Associated with Perceived Benefits of Caregiving for People with Heart Failure. J. Cardiovasc. Nurs..

[19] Arapi A., Vellone E., Ivziku D., Duka B., Taci D., Notarnicola I., Stievano A., Prendi E., Rocco G., De Maria M. (2023). Psychometric Characteristics of the Self-Care of Chronic Illness Inventory in Older Adults Living in a Middle-Income Country. Int. J. Environ. Res. Public Health.

[20] Pucciarelli G., Buck H.G., Barbaranelli C., Savini S., Simeone S., Juarez-Vela R., Alvaro R., Vellone E. (2016). Psychometric Characteristics of the Mutuality Scale in Stroke Patients and Caregivers. Gerontologist.

[21] Bassola B., Cilluffo S., Bolgeo T., Simonelli N., Di Matteo R., Dal Molin A., Rasero L., Vellone E., Lusignani M., Iovino P. (2025). Psychometric Testing of the Mutuality Scale in Patients and Caregiver Dyads After the Onset of Coronary Heart Disease. Res. Nurs. Health.

[22] Golics C.J., Basra M.K., Salek M.S., Finlay A.Y. (2013). The impact of patients’ chronic disease on family quality of life: An experience from 26 specialties. Int. J. Gen. Med..

[23] Murphy A., McGowan C., McKee M., Suhrcke M., Hanson K. (2019). Coping with healthcare costs for chronic illness in low-income and middle-income countries: A systematic literature review. BMJ Glob. Health.

[24] International Labour Organization (2022). Long-Term Care for the Elderly in Albania: Challenges and Key Policy Issues.

[25] De Maria M., Vellone E., Ausili D., Alvaro R., Di Mauro S., Piredda M., De Marinis M., Matarese M. (2019). Self-care of patient and caregiver DyAds in multiple chronic conditions: A LongITudinal studY (SODALITY) protocol. J. Adv. Nurs..

[26] Gagnier J.J., Lai J., Mokkink L.B., Terwee C.B. (2021). COSMIN reporting guideline for studies on measurement properties of patient-reported outcome measures. Qual. Life Res..

[27] Kline R.B. (2023). Principles and Practice of Structural Equation Modeling.

[28] Wild D., Grove A., Martin M., Eremenco S., McElroy S., Verjee-Lorenz A., Erikson P. (2005). Principles of Good Practice for the Translation and Cultural Adaptation Process for Patient-Reported Outcomes (PRO) Measures: Report of the ISPOR Task Force for Translation and Cultural Adaptation. Value Health.

[29] Aderaj S., Arapi A., Mazzotta R., Stievano A., Taci D., Ivziku D., Bernalte-Marti V., Vellone E., Rocco G., De Maria M. (2025). Caregiver Contribution to Self-Care of Chronic Illness Inventory: Evaluation of Measurement Properties in a Middle-Income Country. Nurs. Rep..

[30] Barbaranelli C., Lee C.S., Vellone E., Riegel B. (2015). The problem with Cronbach’s Alpha: Comment on Sijtsma and van der Ark (2015). Nurs. Res..

[31] van der Linden W.J. (2016). Handbook of Item Response Theory: Three Volume Set.

[32] Muthén L.K., Muthén B.O. (2012). Mplus User’s Guide.

[33] Dellafiore F., Chung M.L., Alvaro R., Zeffiro V., Ercole V., Pucciarelli G. (2022). Influence of mutuality on quality of life in heart failure patient with inadequate self-care and caregiver dyads: An actor-partner interdependence model analysis. Eur. J. Cardiovasc. Nurs..

[34] Meade A.W., Johnson E.C., Braddy P.W. (2008). Power and sensitivity of alternative fit indices in tests of measurement invariance. J. Appl. Psychol..

[35] Hu L., Bentler P.M. (1999). Cutoff criteria for fit indexes in covariance structure analysis: Conventional criteria versus new alternatives. Struct. Equ. Model. Multidiscip. J..

[36] Raykov T., Marcoulides G.A. (2008). An Introduction to Applied Multivariate Analysis.

[37] Jöreskog K.G., Sörbom D. (1993). LISREL 8: Structural Equation Modeling with the SIMPLIS Command Language.

[38] Tabachnick B.G., Fidell L.S. (2013). Using Multivariate Statistics.

[39] Bollen K.A., Long J.S., Bollen K.A., Long J.S. (1993). Testing Structural Equation Models.

[40] Barbaranelli C., Lee C.S., Vellone E., Riegel B. (2014). Dimensionality and reliability of the self-care of heart failure index scales: Further evidence from confirmatory factor analysis. Res. Nurs. Health.

[41] Fornell C., Larcker D.F. (1981). Evaluating Structural Equation Models with Unobservable Variables and Measurement Error.

[42] Raykov T. (2012). Scale construction and development using structural equation modeling. Handbook of Structural Equation Modeling.

[43] Bagozzi R.P. (1983). Issues in the Application of Covariance Structure Analysis: A Further Comment. J. Consum. Res..

[44] Nunnally J.C., Bernstein I.H. (1994). Psychometric Theory.

[45] Brown J.D. (1999). Statistics Corner Questions and Answers About Language Testing Statistics: Standard Error vs. Standard Error of Measurement. Shiken JALT Test. Eval. SIG Newsl..

[46] Kelley H.H., Thibaut J. (1978). Interpersonal Relations: A Theory of Interdependence.

[47] Lyons K.S., Lee C.S. (2018). The Theory of Dyadic Illness Management. J. Fam. Nurs..

[48] Cohen J. (1988). Statistical Power Analysis for the Behavioral Sciences.

[49] Chen C., Zhao Q., Zhang X., Yang Q., Dong X., Zhang Y., Fan X. (2022). The relationship between mutuality and contributions to self-care in family caregivers of patients with heart failure: Multiple mediating effects of resilience and self-efficacy. Eur. J. Cardiovasc. Nurs..

[50] Mokkink L.B., Terwee C.B., Patrick D.L., Alonso J., Stratford P.W., Knol D.L., Bouter L.M., de Vet H.C.W. (2010). The COSMIN checklist for assessing the methodological quality of studies on measurement properties of health status measurement instruments: An international Delphi study. Qual. Life Res..

[51] Weijters B., Geuens M., Schillewaert N. (2009). The proximity effect: The role of inter-item distance on reverse-item bias. Int. J. Res. Mark..

[52] Streck B.P., Wardell D.W., Wood G.L. (2020). Family Caregiver-Receiver Mutuality: A Concept Analysis. Adv. Nurs. Sci..

[53] Billiet J., Loosveldt G. (1988). Improvement of the Quality of Responses to Factual Survey Questions by Interviewer Training. Public Opin. Q..

[54] de Leeuw E.D. (2001). Reducing Missing Data in Surveys: An Overview of Methods. Qual. Quant..

