# Supplementary material for: Psychometric Evaluation of the Mutuality Scale in Older Adults with Multiple Chronic Conditions and Their Caregivers Living in a Low–Middle-Income Country
Source: Nurs Rep. 2026 Aug 25;16(9):297. doi: 10.3390/nursrep16090297 (PMC13610364; doi:10.3390/nursrep16090297)
Supplement: Supplementary file 1 [file nursrep-16-00297-s001.zip › nursrep-4334849-supplementary.pdf]

Table S1. Bootstrap confidence intervals (5,000 resamples) for the standardized factor loadings and residual covariances of the final Mutuality Scale models (patients and caregivers).

		Patients			Caregivers		
**Factor/Parameter**	**Item**	**Loading**	**SE**	**95% CI**	**Loading**	**SE**	**95% CI**
Love and affection	2	0.844	0.024	0.793–0.887	0.824	0.025	0.774–0.873
	5	0.876	0.02	0.833–0.914	0.851	0.042	0.757–0.922
	8	0.857	0.021	0.811–0.893	0.819	0.025	0.767–0.863
Shared activities	3	0.721	0.032	0.655–0.778	0.755	0.024	0.706–0.801
	7	0.810	0.021	0.766–0.849	0.823	0.020	0.783–0.860
	11	0.816	0.021	0.771–0.854	0.820	0.020	0.778–0.856
	14	0.821	0.018	0.783–0.854	0.849	0.017	0.814–0.880
Shared values	1	0.677	0.042	0.589–0.756	0.647	0.042	0.567–0.729
	9	0.786	0.032	0.717–0.846	0.758	0.032	0.695–0.816
Reciprocity	4	0.799	0.025	0.747–0.843	0.653	0.033	0.587–0.715
	6	0.723	0.029	0.662–0.776	0.733	0.026	0.681–0.783
	10	0.783	0.026	0.728–0.831	0.816	0.020	0.777–0.855
	12	0.769	0.03	0.705–0.822	0.751	0.026	0.697–0.798
	13	0.841	0.019	0.802–0.876	0.825	0.020	0.786–0.864
	15	0.791	0.021	0.749–0.830	0.735	0.028	0.678–0.789
Mutuality scale	Love	0.814	0.025	0.762–0.860	0.760	0.030	0.700–0.814
	Shared Activities	0.950	0.017	0.915–0.982	0.931	0.016	0.902–0.963
	Shared Values	0.892	0.027	0.836–0.941	0.938	0.027	0.885–0.991
	Reciprocity	0.969	0.014	0.940–0.993	0.995	0.009	0.966–1.000
**Residual covariances**							
	#2–#3	0.296	0.056	0.184–0.404	0.336	0.068	0.196–0.461
	#2–#4	0.200	0.062	0.071–0.314			
	#3–#5				0.246	0.072	0.118–0.402
	#1–#4				0.205	0.052	0.101–0.301
	#11–#15				0.177	0.056	0.066–0.287
	#4–#15				0.345	0.055	0.228–0.440

Note. SE = standard error; CI = confidence interval, bootstrap (5,000 resamples). Values are standardized (STDYX) factor loadings for items within each factor and for first-order factors within the second-order model, and standardized residual covariances for the item pairs listed under "Residual covariances."
